# Causal relationships of grey matter structures in multiple sclerosis and neuromyelitis optica spectrum disorder: insights from Mendelian randomization

**DOI:** 10.1093/braincomms/fcae308

**Published:** 2024-09-11

**Authors:** Jie Sun, Yingying Xie, Tongli Li, Yunfei Zhao, Wenjin Zhao, Zeyang Yu, Shaoying Wang, Yujie Zhang, Hui Xue, Yayuan Chen, Zuhao Sun, Zhang Zhang, Yaou Liu, Ningnannan Zhang, Feng Liu

**Affiliations:** Department of Radiology and Tianjin Key Laboratory of Functional Imaging, Tianjin Medical University General Hospital, Tianjin 300052, China; Department of Radiology, The First Affiliated Hospital of Fujian Medical University, Fujian 350005, China; Department of Radiology and Tianjin Key Laboratory of Functional Imaging, Tianjin Medical University General Hospital, Tianjin 300052, China; Department of Radiology and Tianjin Key Laboratory of Functional Imaging, Tianjin Medical University General Hospital, Tianjin 300052, China; Department of Radiology and Tianjin Key Laboratory of Functional Imaging, Tianjin Medical University General Hospital, Tianjin 300052, China; Department of Radiology and Tianjin Key Laboratory of Functional Imaging, Tianjin Medical University General Hospital, Tianjin 300052, China; Department of Radiology and Tianjin Key Laboratory of Functional Imaging, Tianjin Medical University General Hospital, Tianjin 300052, China; Department of Radiology and Tianjin Key Laboratory of Functional Imaging, Tianjin Medical University General Hospital, Tianjin 300052, China; Department of Radiology and Tianjin Key Laboratory of Functional Imaging, Tianjin Medical University General Hospital, Tianjin 300052, China; Department of Radiology and Tianjin Key Laboratory of Functional Imaging, Tianjin Medical University General Hospital, Tianjin 300052, China; Department of Radiology and Tianjin Key Laboratory of Functional Imaging, Tianjin Medical University General Hospital, Tianjin 300052, China; Department of Radiology and Tianjin Key Laboratory of Functional Imaging, Tianjin Medical University General Hospital, Tianjin 300052, China; Department of Radiology, Beijing Tiantan Hospital, Capital Medical University, Beijing 100070, China; Department of Radiology and Tianjin Key Laboratory of Functional Imaging, Tianjin Medical University General Hospital, Tianjin 300052, China; Department of Radiology and Tianjin Key Laboratory of Functional Imaging, Tianjin Medical University General Hospital, Tianjin 300052, China

**Keywords:** multiple sclerosis, neuromyelitis optica spectrum disorder, Mendelian randomization, cortex, subcortex

## Abstract

Multiple sclerosis and neuromyelitis optica spectrum disorder are two debilitating inflammatory demyelinating diseases of the CNS. Although grey matter alterations have been linked to both multiple sclerosis and neuromyelitis optica spectrum disorder in observational studies, it is unclear whether these associations indicate causal relationships between these diseases and grey matter changes. Therefore, we conducted a bidirectional two-sample Mendelian randomization analysis to investigate the causal relationships between 202 grey matter imaging–derived phenotypes (33 224 individuals) and multiple sclerosis (47 429 cases and 68 374 controls) as well as neuromyelitis optica spectrum disorder (215 cases and 1244 controls). Our results suggested that genetically predicted multiple sclerosis was positively associated with the surface area of the left parahippocampal gyrus (*β* = 0.018, *P* = 2.383 × 10^−4^) and negatively associated with the volumes of the bilateral caudate (left: *β* = −0.020, *P* = 7.203 × 10^−5^; right: *β* = −0.021, *P* = 3.274 × 10^−5^) and putamen nuclei (left: *β* = −0.030, *P* = 2.175 × 10^−8^; right: *β* = −0.024, *P* = 1.047 × 10^−5^). In addition, increased neuromyelitis optica spectrum disorder risk was associated with an increased surface area of the left paracentral gyrus (*β* = 0.023, *P* = 1.025 × 10^−4^). Conversely, no evidence was found for the causal impact of grey matter imaging–derived phenotypes on disease risk in the opposite direction. We provide suggestive evidence that genetically predicted multiple sclerosis and neuromyelitis optica spectrum disorder are associated with increased cortical surface area and decreased subcortical volume in specific regions. Our findings shed light on the associations of grey matter alterations with the risk of multiple sclerosis and neuromyelitis optica spectrum disorder.

## Teaser

Liu *et al*., utilizing a bidirectional two-sample Mendelian randomization analysis, investigated the causal relationships between grey matter structures and the risk of multiple sclerosis and neuromyelitis optica spectrum disorder. Their findings provided novel insights into the complex interplay between grey matter alterations and the risk of these debilitating inflammatory demyelinating diseases.

## Graphical Abstract

**Graphical Abstract:**
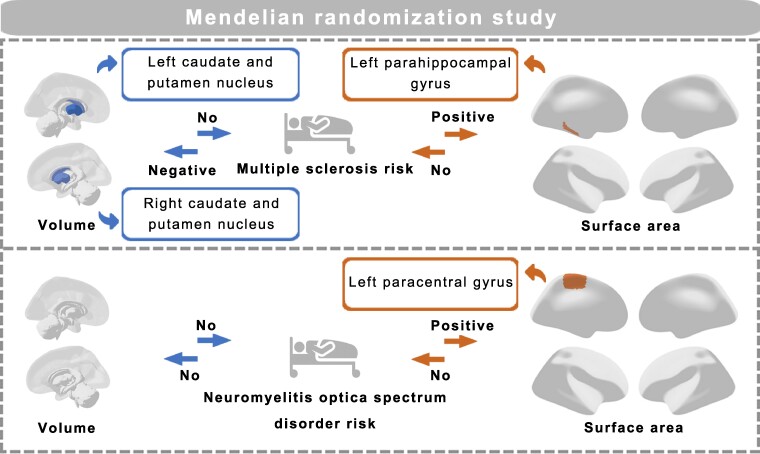

## Introduction

Multiple sclerosis and neuromyelitis optica spectrum disorder (NMOSD) are two common debilitating inflammatory demyelinating diseases of the CNS, both of which are characterized by severe physical and cognitive deficits.^1,2^ Currently, the incidences of multiple sclerosis and NMOSD are increasing worldwide, posing a substantial impact on healthcare systems and leading to an increasing socioeconomic burden, including the costs of disease-modifying therapies (DMTs) and productivity loss.^3,4^ Multiple sclerosis presents a diverse array of clinical and imaging features, a part of which often overlaps with those of NMOSD, posing a challenge in the clinical differentiation between these two conditions. Although serologic testing for aquaporin-4 (AQP4) antibody has high specificity, it may yield negative results in a subset of patients with NMOSD.^2^ Numerous observational studies have been conducted to distinguish between these two diseases,^5,6^ and neuroimaging has been proven valuable for supporting differential diagnosis. Consequently, there is an urgent need to investigate the underlying neurobiological mechanisms of multiple sclerosis and NMOSD.

Grey matter (GM), which is critical to the CNS, has emerged as pivotal in unravelling the complex neurobiological mechanisms underlying multiple sclerosis and NMOSD. Recent research has emphasized the profound impact of these conditions on GM, its susceptibility to immune-mediated processes and neuroinflammation.^7^ Moreover, observational studies have extensively investigated the relationship between GM alterations and the onset of these two diseases. In patients with multiple sclerosis, the GM volume (GMV) exhibits widespread atrophy, primarily affecting the thalamus, while in patients with NMOSD, atrophy is more confined to the hippocampus and visual cortex.^5,8^ The GMV changes have been reported to lead to both physical disability and cognitive dysfunction and were evaluated as a potential clinical trial end-point because they are considered sensitive markers for the response to DMTs.^9,10^

However, the exact causal relationships between these two diseases and GM damage remain uncertain. Whether GM alteration serves as a risk factor or a secondary change in the context of multiple sclerosis and NMOSD is not fully understood. As an important consequence of neuroaxonal loss, GM alterations are the most commonly used imaging biomarker for quantifying neurodegeneration in multiple sclerosis and NMOSD clinical trials.^11^ Nevertheless, GM alterations are independent of disease activity^10,12^ and may appear before the occurrence of multiple sclerosis and NMOSD. Moreover, the interaction between disease-specific and age-related volume changes remains complex in patients with multiple sclerosis and NMOSD,^5,13^ as GM atrophy is attributable to increased brain ageing and decreased disease pathology over time. In addition, some results obtained from the observational studies of patients with multiple sclerosis and NMOSD appear inconsistent, possibly due to some potential confounding factors such as differences in the population, sample size, assessment methods, covariates or clinical factors.^14,15^ Therefore, it is crucial to clarify the associations between GM structures and these two diseases.

Mendelian randomization (MR) is a powerful analytic approach that enables the estimation of causal relationships using epidemiological data.^16^ This method leverages single-nucleotide polymorphisms (SNPs) or genetic variants as instrumental variables (IVs) for causal inference and is grounded in Mendel’s laws of inheritance.^17^ The inherent directionality and stability of these genetic variants make the associations between exposures and outcomes derived from MR less susceptible to reverse causality bias or potential confounding factors than those derived from observational studies. To date, the MR method has been used to successfully establish causal relationships between intricate exposures and their corresponding outcomes.^18,19^ In this study, we attempt to assess the causal relationships between these two demyelinating diseases and GM morphology through bidirectional two-sample MR analyses.

## Materials and methods

### Data sources

We utilized summary statistics from genome-wide association studies (GWASs) for imaging-derived phenotypes (IDPs) sourced from the UK Biobank, which can be accessed at https://open.win.ox.ac.uk/ukbiobank/big40/. This data set encompassed data from 33 224 individuals of European ancestry (15 813 males and 17 411 females),^20^ and brain GM–related phenotypes were obtained. In this study, a total of 202 cortical and subcortical GM–related IDPs sourced from the UK Biobank were employed. Specifically, we included a total of 186 cortical measurements, comprising 62 surface area measurements, 62 cortical thickness measurements and 62 volume measurements, in addition to 16 subcortical volume measurements. For more comprehensive details about these IDPs, please refer to the online reference provided by the UK Biobank at https://biobank.ctsu.ox.ac.uk/crystal/crystal/docs/brain_mri.pdf. Furthermore, the International Multiple Sclerosis Genetics Consortium^21^ and Estrada *et al*.^22^ provided us with GWAS summary statistics for multiple sclerosis [47 429 cases (16 757 males and 30 672 females) and 68 374 controls (25 650 males and 42 724 females)] and NMOSD [215 cases and 1244 controls (the sex ratio was not mentioned)], respectively. Notably, all the data related to patients with multiple sclerosis and NMOSD were derived from individuals of European ancestry, and there was no sample overlap between the GM IDPs and the multiple sclerosis or NMOSD data.

### Selection of genetic instruments

The selection of IVs in each pair of MR analyses should satisfy three assumptions: (i) SNPs were strongly associated with exposure, (ii) SNPs were not associated with outcome and (iii) SNPs were not associated with confounding factors. To select the eligible instruments, the significance threshold for GWAS summary statistics was set at *P* < 5 ×10^−8^ for both multiple sclerosis and all GM IDPs. However, due to the smaller sample size,^22^ a *P*-value was set at 5 ×10^−6^ for the NMOSD trait.^23^ To account for linkage disequilibrium (LD), a clumping algorithm was employed with an *r*^2^ cut-off of 0.001 and a window size of 1 Mb for both diseases, with the European template from the 1000 Genomes Project serving as the LD reference. Subsequently, the exposure and outcome data were harmonized according to the same effect alleles, and palindromic SNPs were excluded. If the IVs were not available in the outcome data set, we used the proxy SNP with *r*^2^ > 0.8 instead.^24^ Then, we used the RadialMR package to remove heterogeneous SNPs (https://github.com/WSpiller/RadialMR/).^25^ The *F*-statistics for each IV were computed to evaluate the power of the IVs. To mitigate the impact of weak instrument bias, IVs with *F*-statistics exceeding 10 were selected for further analyses.^26^

### Bidirectional two-sample MR analyses

Bidirectional two-sample MR analyses were conducted to investigate the causal relationships between GM structure phenotypes and the two demyelinating diseases. In the MR analysis for causal estimation of diseases affecting GM IDPs, the two demyelinating diseases were used as exposures, with GM IDPs as outcomes. Conversely, in the MR analysis for causal estimation of GM IDPs on diseases, GM IDPs were employed as exposures, with the two demyelinating diseases as outcomes. The inverse-variance weighted (IVW) method^27^ was implemented as a primary approach, which assumes that all IVs are valid and provides a precise estimate if the core assumption of MR is not violated. The significance level was set as Bonferroni-corrected *P* < 0.05/202 = 2.48 × 10^−4^. To validate our results, four additional MR methods were employed. First, the MR robust adjusted profile score (MR-RAPS) method was utilized to account for both systematic and idiosyncratic pleiotropy, thereby enabling robust inference for the MR analysis involving numerous weak instruments.^28^ Second, the weighted median method was employed to provide dependable estimates, even in scenarios where up to 50% of the IVs might be invalid, by allowing half of the SNPs to serve as valid instruments for causal estimation.^29^ Third, the weighted mode method was applied to yield consistent estimates by reducing bias and minimizing the Type I error rate when the relaxed IV assumption was met.^30^ Finally, the MR-Egger method estimated the causal effect through the slope coefficient of the Egger regression, with the intercept being used to detect the presence of directional pleiotropy, thereby improving the robustness of the estimate, even when none of the IVs were invalid.^31^ Notably, the Wald ratio method was used when only one genetic instrument was available.^32^ In the present study, the MR analyses were conducted in accordance with the STROBE-MR checklist^33^ and guidelines for performing MR investigations.^34^

### Sensitivity analysis

A range of sensitivity analyses were carried out to verify our putative causalities obtained with bidirectional MR. First, heterogeneity within the IVs was assessed using Cochran’s *Q* statistic for IVW analyses^35^ and Rücker’s *Q* statistic for MR-Egger analyses,^36^ respectively. Second, the MR pleiotropy residual sum and outlier (MR-PRESSO) global test was utilized to detect horizontal pleiotropy (https://github.com/rondolab/MR-PRESSO/).^37^ Third, MR-Egger regression was used to assess the mean pleiotropic effects of all genetic variants. Evidence of horizontal pleiotropy was indicated when MR-Egger intercepts for all associations differed from zero (*P* < 0.05).^31^ Finally, a leave-one-out analysis was performed to ascertain whether a single SNP was responsible for driving the primary causal relationship.

### Statistical analysis

We determined the statistical power for each MR test using an online power calculator (https://sb452.shinyapps.io/power/). Adequate statistical power was defined as a value >80%.

**Figure 1:**
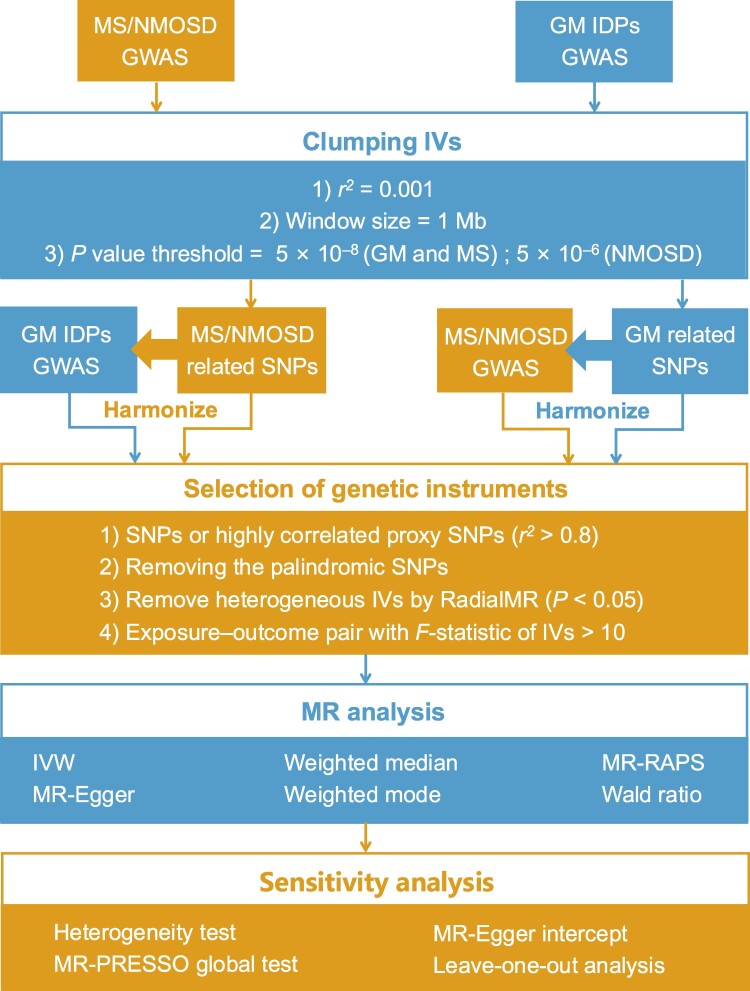
**A flow chart of the causal inference between GM IDPs and demyelinating diseases.** First, to select the eligible instruments, the significance threshold for GWAS summary statistics was set at *P* < 5 ×10^−8^ for both multiple sclerosis and all GM IDPs and *P* < 5 ×10^−6^ for the NMOSD trait. Second, to account for LD, a clumping algorithm was employed with an *r*^2^ cut-off of 0.001 and a window size of 1 Mb. Third, the exposure and outcome data were harmonized according to the same effect alleles, and palindromic SNPs were excluded. If the IVs were not available in the outcome data set, we used the proxy SNP with *r*^2^ > 0.8 instead. Then, we used the RadialMR package to remove heterogeneous SNPs. IVs with *F*-statistics exceeding 10 were selected for further analyses. Fourth, the IVW method was implemented as a primary approach, and other four additional MR methods were employed (MR-RAPS, weighted median, weighted mode and MR-Egger). The Wald ratio method was used when only one genetic instrument was available. Last, a range of sensitivity analyses were carried out, including a heterogeneity test, MR-PRESSO global test, MR-Egger regression analysis and leave-one-out analysis.

All the analyses mentioned above were conducted using the open-source statistical software *R* (version 4.2.2). TwoSampleMR (version 0.5.6), RadialMR (version 1.0), MRPRESSO (version 1.0) and mr.raps (version 0.4.1) were used for MR analyses. The study design is shown in Fig. 1.

## Results

### The causal effects of multiple sclerosis and NMOSD on GM IDPs

**Figure 2:**
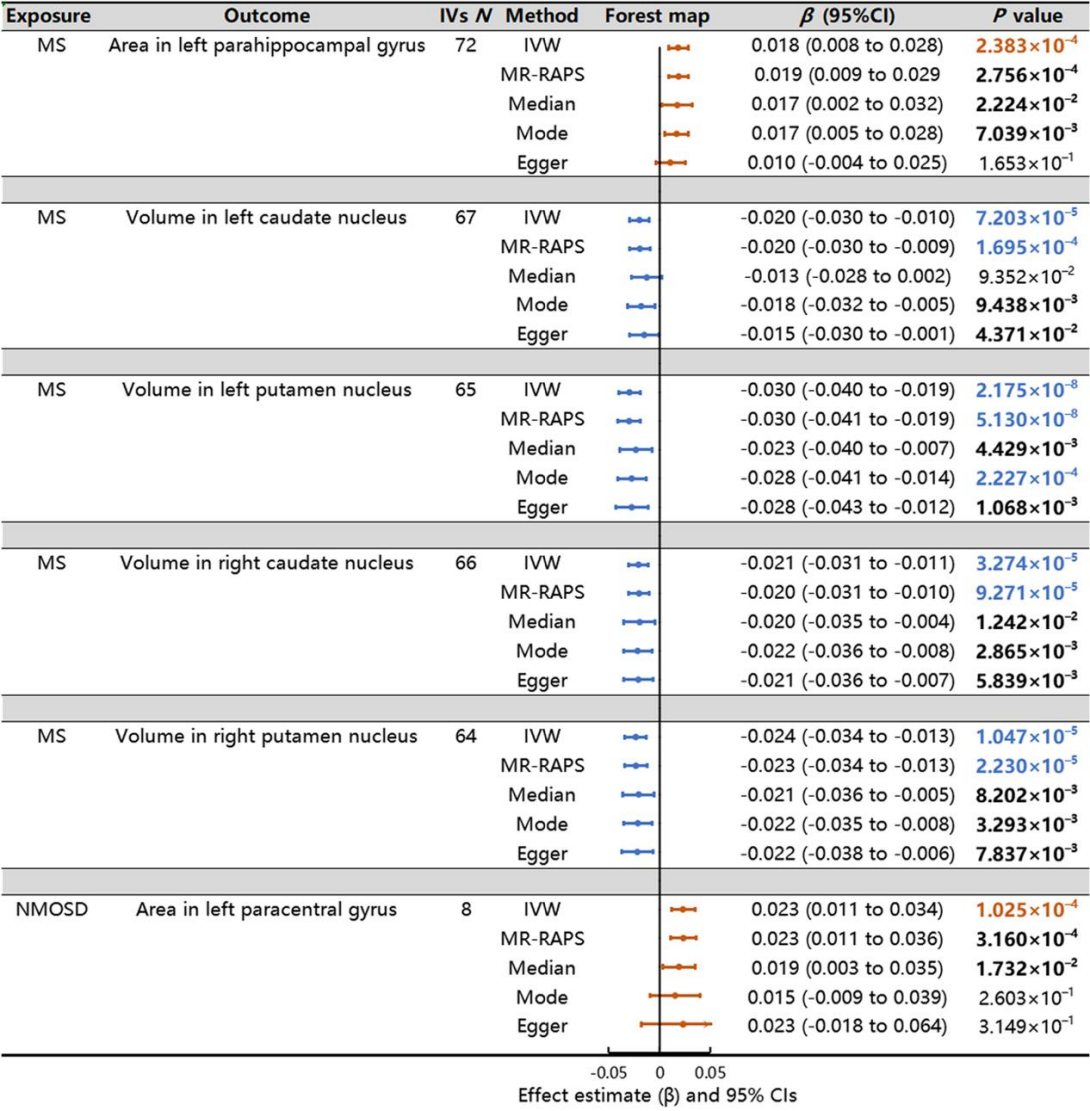
**MR estimates from two demyelinating diseases on GM phenotypes.** Causal effects were estimated using five two-sample MR methods: IVW, MR-RAPS, weighted median, weighted mode and MR-Egger. The IVW method assumes that all the IVs are valid. The MR-RAPS method is used to account for both systematic and idiosyncratic pleiotropy, although for weak instruments. The weighted median method provides dependable estimates, even in scenarios where up to 50% of the IVs might be invalid. The weighted mode method yields consistent estimates by reducing bias and minimizing the Type I error rate. The MR-Egger method estimates the causal effect through the slope coefficient of the Egger regression. The forest plot illustrates the significant causalities. The effect estimates represent a change in GM IDPs per unit change in demyelinating diseases, and the error bars represent 95% CI. All statistical tests were two-sided. *P*-value <0.05 is represented in bold, and *P*-value < 2.48 × 10^−4^ is represented in colour under the Bonferroni correction. The arrows indicate that the maximum interval on the *x-*axis is extended.

**Figure 3:**
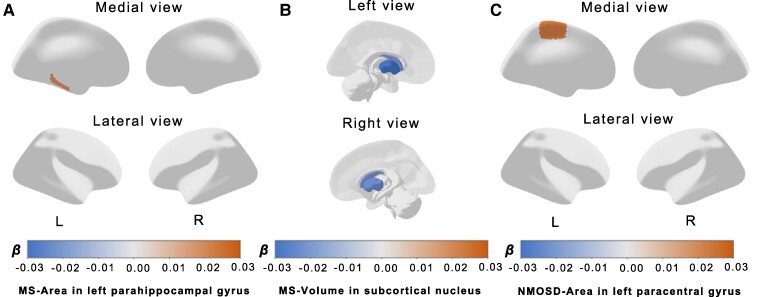
**The brain anatomical region of the significant statistical results.** The pattern diagrams display the brain anatomical region of the significant statistical results by IVW methods (*P* < 2.48 × 10^−4^) between risk of multiple sclerosis (47 429 cases and 68 374 controls) or NMOSD (215 cases and 1244 controls) and GM IDPs (33 224 individuals). Our results suggest that genetically predicted multiple sclerosis was positively associated with the surface area of the left parahippocampal gyrus (**A**) and negatively associated with the volume of the bilateral caudate and putamen nuclei (**B**). A higher NMOSD risk was associated with the increased surface area of the left paracentral gyrus (**C**). The orange represents a positive effect of *β*, while the blue represents a negative effect of *β*. L, left; MS, multiple sclerosis; R, right.

Outlier IVs detected by RadialMR were removed from the MR analysis (Supplementary Table 1). The full lists of selected IVs are provided in Supplementary Table 2. The *F*-statistic values were all ≥10 (Supplementary Table 3). The full results of causal estimates for multiple sclerosis risk on GM IDPs are given in Supplementary Table 4, and the significant causal effects of multiple sclerosis on GM IDPs after adjusting for multiple comparisons are shown in Fig. 2. Specifically, a heightened risk of multiple sclerosis was associated with an increased surface area of the left parahippocampal gyrus (IVW *β* = 0.018, 95% CI: 0.008–0.028, *P* = 2.383 × 10^−4^, Fig. 3A) and reductions in subcortical volume in various regions, including the left caudate nucleus (IVW *β* = −0.020, 95% CI: −0.030 to −0.010, *P* = 7.203 × 10^−5^, Fig. 3B), left putamen nucleus (IVW *β* = −0.030, 95% CI: −0.040 to −0.019, *P* = 2.175 × 10^−8^, Fig. 3B), right caudate nucleus (IVW *β* = −0.021, 95% CI: −0.031 to −0.011, *P* = 3.274 × 10^−5^, Fig. 3B) and right putamen nucleus (IVW *β* = −0.024, 95% CI: −0.034 to −0.013, *P* = 1.047 × 10^−5^, Fig. 3B). Additionally, as shown in Fig. 2 and Supplementary Table 4, we observed that an elevated risk of NMOSD was associated with an increase in the surface area of the left paracentral gyrus (IVW *β* = 0.023, 95% CI: 0.011–0.034, *P* = 1.025 × 10^−4^, Fig. 3C). The forest plots illustrating these MR estimates for the effects of SNPs associated with demyelinating diseases on GM IDPs are shown in Fig. 2. In addition, all SNPs associated with both demyelinating diseases and GM structures are shown in Supplementary Fig. 1, with coloured lines representing the slopes of different regression analyses. The statistical power of all associations is also shown in Supplementary Table 5 under significance levels of 0.05 and 2.48 × 10^−4^.

### The causal effects of GM IDPs on multiple sclerosis and NMOSD

Outlier IVs detected by RadialMR were removed from the MR analysis (Supplementary Table 6). The full lists of selected IVs are provided in Supplementary Table 7. The *F*-statistic values were all ≥10 (Supplementary Table 8). There were no significant causal effects of GM IDPs on demyelinating diseases according to the Bonferroni correction (Supplementary Table 9).

### Sensitivity analyses

Sensitivity analyses were conducted to validate our putative causal relationships. First, no significant evidence of heterogeneity was detected from the Cochran’s *Q* and Rucker’s *Q*′ statistics, respectively (Supplementary Table 5). Second, no signs of horizontal pleiotropy were detected through the MR-PRESSO global test and the MR-Egger regression (Supplementary Table 5). Third, the leave-one-out analysis showed that no single SNP drove the causal estimates (Supplementary Table 10), and no asymmetry was detected in the funnel plot (Supplementary Fig. 2). Fourth, when employing the same threshold (*P* < 2.48 × 10^−4^), the number of associations supported by the IVW was 6, while the numbers of associations supported by the MR-RAPS, weighted median, weighted mode and MR-Egger methods were only 4, 0, 1 and 0, respectively (Fig. 2A; Supplementary Table 4). These discrepancies may have arisen from the lower statistical power of these four methods compared with that of the IVW method.^30^ However, the directions of the statistically significant causal relationships in the other MR methods were consistent with those in the IVW method, and the numbers were 6, 5, 5 and 4, respectively, when the threshold changed to 0.05. Overall, the sensitivity analyses confirmed the reliability of our putative causal effects on the MR results.

## Discussion

In the present study, we investigated the causal relationships between 202 GM-related IDPs (cortical surface area, cortical thickness, and cortical and subcortical volumes) and two inflammatory demyelinating diseases (multiple sclerosis and NMOSD). We identified significant causal effects of multiple sclerosis on five GM IDPs and of NMOSD on one GM IDP. To the best of our knowledge, this study represents the first attempt to investigate the causal relationships between GM IDPs and multiple sclerosis as well as NMOSD.

We found a causal association between genetically predicted multiple sclerosis risk and increased surface area in the left parahippocampal gyrus. Previous observational studies have not found any differences between patients with multiple sclerosis and healthy controls (HCs) in the surface area of the parahippocampal gyrus,^38^ but the injury to the parahippocampal gyrus plays an important role in patients with multiple sclerosis. First, the lesions in the parahippocampal gyrus are associated with bowel and urinary incontinence in patients with multiple sclerosis^39,40^ and may cause significant structural changes in the morphology of the parahippocampal gyrus, which manifests as increased surface area. Second, patients with multiple sclerosis demonstrated diverse abnormal functional connections in the parahippocampal gyrus,^41,42^ and the increased surface area in patients with multiple sclerosis may reflect the structural correlates of this functional adaptation.^43^ Finally, damage to the parahippocampal gyrus has been linked to cognitive decline and disability in patients with multiple sclerosis^44,45^ and notably improved after cognitive therapy and motor rehabilitation.^46,47^ Thus, our results suggest that the parahippocampal gyrus is a potential target for therapeutic interventions, because it is a sensitive site that is significantly affected by multiple sclerosis risk. However, a decreased volume of the parahippocampal gyrus was identified in previous observational studies.^48-50^ On the one hand, atrophy of the parahippocampal gyrus may manifest as a reduction in thickness rather than in surface area.^44^ On the other hand, observational studies often struggle to fully address confounding factors, such as the influence of ageing, or environmental factors.^51^ In addition, previous research has speculated that cortical reorganization in adjacent brain regions induced by heightened cortical networks, or as an adaptive mechanism to compensate for declining cognitive performance, could lead to compensatory brain hypertrophy, consequently increasing cortical volume.^52^ Thus, multiple sclerosis may not be limited to the pattern of GM atrophy alone; it can also include a compensatory increased in the GM pattern. In summary, our findings contribute to additional evidence of the association between the parahippocampal gyrus and multiple sclerosis, but further analysis is needed to fully elucidate the exact patterns of surface area damage in the parahippocampal gyrus.

We observed causal relationships between genetically predicted multiple sclerosis and decreased volumes of the bilateral caudate and putamen nuclei, findings that were in line with the results of previous observational studies and meta-analyses.^53-55^ The caudate and putamen nuclei exhibited diverse patterns of reduction in patients with different multiple sclerosis subtypes: atrophy in the early stage of relapsing–remitting multiple sclerosis (RRMS) is mainly determined by global lesion burden, while delayed atrophy in primary-progressive multiple sclerosis (PPMS) is mainly explained by local microstructural damage and susceptibility changes.^56,57^ Combined with the diverse GM injury patterns and mechanisms between multiple sclerosis phenotypes identified in previous observational studies, our findings further confirmed the causal relationship between multiple sclerosis risk and caudate and putamen GMV atrophy. Caudate and putamen atrophy were not only highly correlated with fatigue symptoms^58,59^ but also possibly caused different cognitive phenotypes. Compared with HCs, patients with multiple sclerosis with mild-verbal memory/semantic fluency were characterized by a reduction in putamen volume, and patients with mild-multidomain fluency had a lower caudate volume.^60^ In addition, as the promising imaging biomarkers in multiple sclerosis, the progression of caudate and putamen atrophy decreased after treatment.^61^ In summary, our findings enhance the understanding of the associations between the caudate and putamen nuclei and multiple sclerosis and suggest possible future directions for therapy.

Additionally, we revealed a causal association between NMOSD risk and increased surface area of the left paracentral gyrus. Unfortunately, the structural morphology of the paracentral gyrus in relation to NMOSD has yet to be explored in current observational studies, possibly because of the lower prevalence of and limited research on NMOSD.^62^ Nevertheless, previous studies have indicated that brain activation in the left paracentral gyrus, which is associated with olfactory function, is attenuated in individuals with NMOSD.^63^ NMOSD-specific antibodies target the water channel AQP4 and result in astrocyte damage.^2^ The fine structure of astrocytes is highly plastic and activity dependent;^64^ thus, changes in the surface area of the paracentral lobule may be a hallmark of reactive astrogliosis after brain function impairment. In summary, our study provides new evidence for the link between NMOSD and the paracentral gyrus and highlights the need for further research.

There were no significant causal effects of GM IDPs on the risk of multiple sclerosis or NMOSD. Our findings indicated that GM alterations represent secondary changes rather than risk factors for multiple sclerosis and NMOSD. Therefore, interventions targeting GM alterations in patients with multiple sclerosis and NMOSD may need to be carefully considered. Nevertheless, based on these findings, we cannot rule out the possibility that GM changes arbitrarily mediate the risk of multiple sclerosis and NMOSD. More granular and specific metrics may be necessary to capture the neural basis of GM. Moreover, several observational studies have reported a reduction in GMV in the thalamus.^5,8^ However, this finding was not reflected in our results. There are several potential explanations for this discrepancy. First, previous cross-sectional and longitudinal studies have revealed that decreased white matter (WM) integrity is associated with thalamic atrophy,^57,65^ but association between thalamic lesion and thalamic atrophy is lacking.^66^ Consequently, thalamic atrophy may be secondary to retrograde Wallerian degeneration resulting from WM damage,^67^ rather than attributed to primary inflammatory demyelinating changes. Second, the thalamus exhibits various atrophy patterns among patients with different multiple sclerosis subtypes. Thalamic atrophy is observed initially in patients with PPMS but later in patients with RRMS and secondary-progressive multiple sclerosis.^56^ Third, the rate of thalamic volume loss varies throughout the disease course and is driven by damage accumulation.^68^ In summary, the intricate pathological changes in the thalamus may contribute to the complexity of the results of causal analysis.

There were some limitations in our study that should be considered. First, the number of patients with NMOSD included in GWASs is limited, and in the MR analysis for causal estimation of the effect of the GM structure on NMOSD risk, the genome-wide significance threshold was set at *P* < 5 × 10^−6^ due to the lack of availability of significant SNPs, which may be accompanied by increased pleiotropy and weak instrument bias. However, we made an attempt to overcome this limitation and assess the predisposition to bias due to weak instruments by assessing a variety of sensitivity methods and *F*-statistics. Second, the weak statistical power of our results may be due to other confounding effects or complex clinical subtypes of the two diseases covered in this study. Nevertheless, our significant causal association could provide a genetic perspective for understanding these two diseases and a basis for future in-depth research by using possible subtype GWAS data. Third, because the population we used was composed of Europeans, our results may not be generalizable to other ethnicities and races due to differences in LD structures and allele frequencies. Fourth, due to the varying power of GWAS data for different demyelinating diseases, cross-sectional comparisons could not be made. Hence, the estimated MR effect size must be interpreted with caution when applied to clinical interventions. Finally, our results need to be further verified with specific genetic variants and GM alterations in patients.

## Conclusion

We provided suggestive evidence supporting causal relationships between genetically predicted multiple sclerosis and increased surface area of the left parahippocampal gyrus and decreased volume of the bilateral caudate and putamen nuclei. Additionally, genetically predicted NMOSD was found to be causally associated with increased surface area in the left paracentral gyrus. These findings shed light on the associations between GM alterations and the onset of multiple sclerosis and NMOSD, emphasizing the need for further investigations into the biological functions of these specific brain regions.

## Supplementary Material

fcae308_Supplementary_Data

## Data Availability

The GWAS summary of human GM analysed in the current study is available from the UK Biobank (https://open.win.ox.ac.uk/ukbiobank/big40/). The GWAS summary of multiple sclerosis analysed in this study is available from the International Multiple Sclerosis Genetics Consortium (http://imsgc.net/; https://gwas.mrcieu.ac.uk/). The GWAS summary of NMOSD analysed in this study is available from the NHGRI-EBI GWAS Catalog (https://www.ebi.ac.uk/gwas/downloads/summary-statistics). The codes used to run the analyses described in this study are available at the online repository (https://zenodo.org/records/11111898).
