# Supplementary material for: Causal relationships of grey matter structures in multiple sclerosis and neuromyelitis optica spectrum disorder: insights from Mendelian randomization
Source: Brain Commun. 2024 Sep 11;6(5):fcae308. doi: 10.1093/braincomms/fcae308 (PMC11420985; doi:10.1093/braincomms/fcae308)
Supplement: fcae308_Supplementary_Data [file fcae308_supplementary_data.zip › Supplementary Materials.pdf]

Supplementary Materials

Supplementary Figures

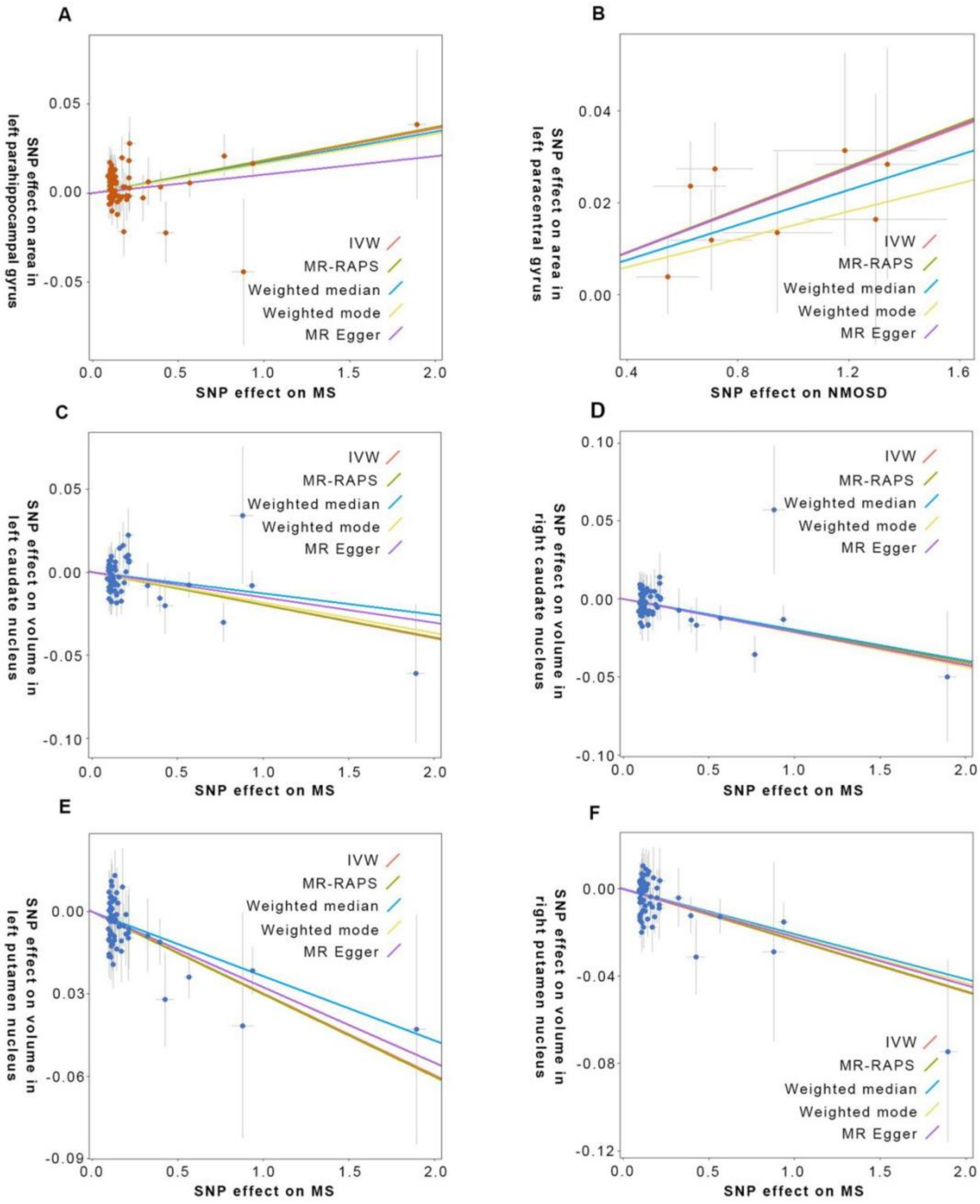

Supplementary Figure 1. MR plots for the relationship of demyelinating diseases and GM phenotypes. (A) Scatter plot of SNP effects of MS on area in left parahippocampal gyrus. (B) Scatter plot of SNP effects of NMOSD on volume in left paracentral gyrus. (C) Scatter plot of SNP effects of MS on volume in left caudate nucleus. (D) Scatter plot of SNP effects of MS on volume in right caudate nucleus. (E) Scatter plot of SNP effects of MS on volume in left putamen nucleus. (F) Scatter plot of SNP effects of MS on volume in right putamen nucleus. The data are expressed as raw β values with 95% CIs. Orange dots represents a positive effect of β, while blue dots represents

a negative effect of β.

Abbreviation: GM, gray matter; IVW, inverse-variance weighted; MR, Mendelian randomization; MS, multiple sclerosis; NMOSD, neuromyelitis optica spectrum disorder; MR-RAPS, MR robust adjusted profile score; SNP, single nucleotide polymorphism.

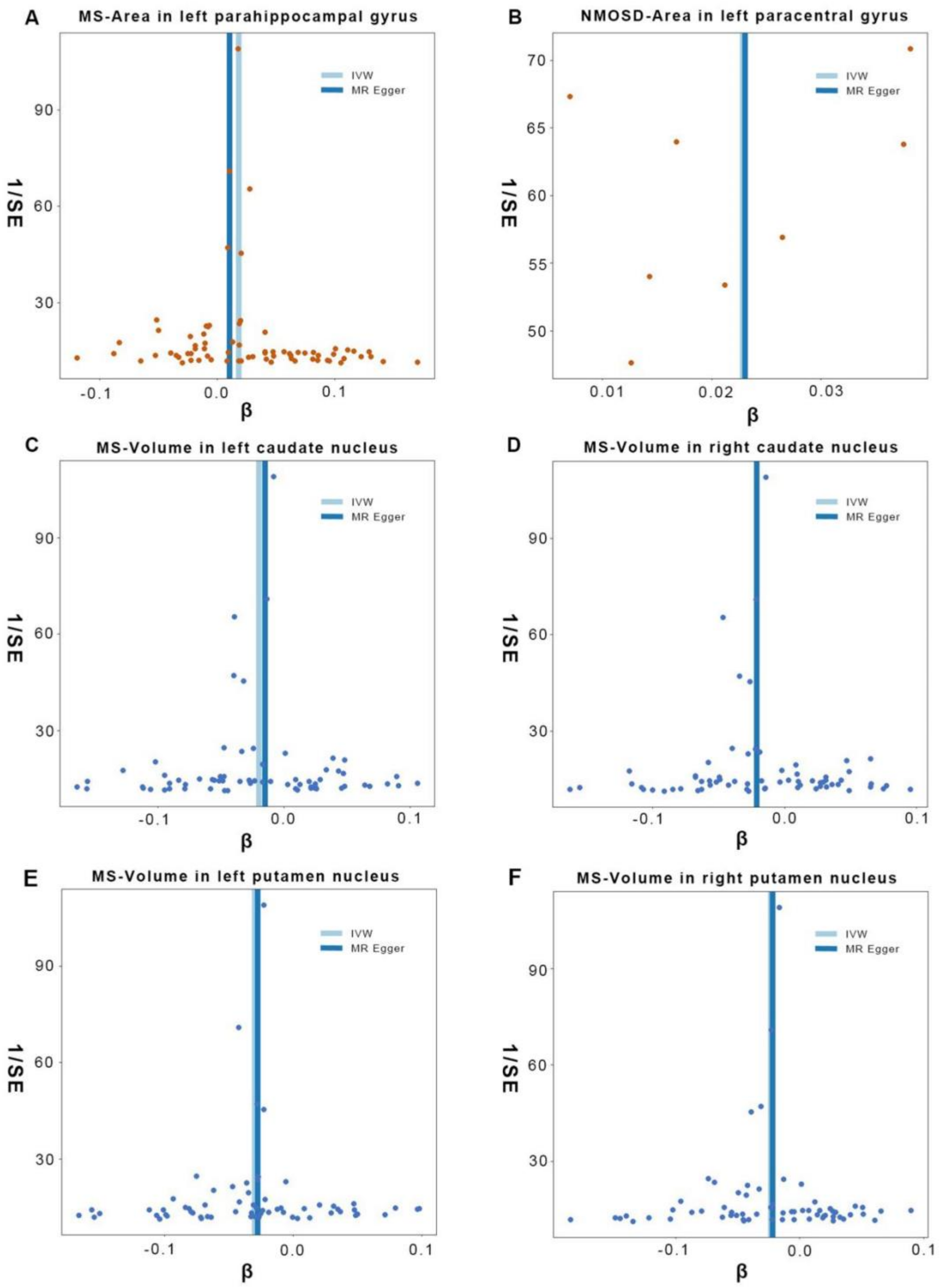

Supplementary Figure 2. Funnel plots for the effect of risk of the demyelinating diseases on GM phenotypes. For each SNP, the resulting MR estimate is plotted against the inverse of the standard error of the MR estimate. Symmetry noted in this plot provides evidence against the presence of directional horizontal pleiotropy. The vertical line represents the summary measure of the effect of MS on area in left parahippocampal gyrus (A); volume in left caudate nucleus (C); volume in left caudate nucleus (D); volume in left putamen nucleus (E); and volume in right putamen nucleus (F) on the log-odds ratio scale. The vertical line also represents the summary measure of the effect of

NMOSD on area in left paracentral gyrus (B). Orange dots represents the effect of β is positive; blue dots represents the effect of β is negative.

Abbreviation: GM, gray matter; IVW, inverse-variance weighted; MR, Mendelian randomization; MS, multiple sclerosis; NMOSD, neuromyelitis optica spectrum disorder; SE, standard error.
